# Re-thinking clinical research training in residency

**Published:** 2014-12-17

**Authors:** Jennifer O’Brien, Marcel D’Eon

**Affiliations:** 1 Department of Anesthesiology, University of Saskatchewan; 2 College of Medicine, University of Saskatchewan

## Abstract

**Background:**

There are good reasons to train clinician researchers, including a lack of translational and patient centered research, a decline in physicians choosing academic careers, the need for physicians who are able to critically appraise research, and accreditation requirements. However, why are we insisting that residents engage in original clinical research?

**Discussion:**

This paper is structured around three questions: 1) Is mandating original research the answer? 2) What ought to be the central purpose of research training? And 3) What are the alternatives to original clinical research? The successful development of clinician-scientists involves many more factors than resident research training. While invoking social accountability and public welfare, we argue for considering the opportunity cost of resident research training. We question the focus on original resident research and challenge medical educators to encourage research training aimed steadfastly at public good in the local setting. Finally, we offer preliminary suggestions for how to move forward.

**Conclusions:**

We conclude that medical educators should critically re-think our programs to develop resident researchers. If it is worthwhile to require original research projects during residency, then we must consider the priorities of local settings to best serve the public interest.

## Introduction

In training residents to be researchers and scholars why are we insisting that they engage in original clinical research? There are good reasons to train clinician researchers, including the obvious and disconcerting void in translational and patient centered research. We are also seeing a decline in physicians choosing academic careers.^1^,^2^ There is a need for physicians out in practice to be able to critically appraise research, and residency program accreditation requirements have traditionally demanded research training.^3^ But why engage in original clinical research projects to address those issues? Although CanMEDS 2015 refocuses the Scholar role to “produce informed research consumers for practice, recognizing that, likely with advanced training, some will choose the option of pursuing roles as clinical investigators or clinician scientists (researchers),”^4^ residency programs tend to follow the example of large academic health centers where residents engage in original clinical research projects. We believe wholeheartedly in evidence-informed medicine, the importance of fostering these skills in residents, and the value of medical research for the public good. However, is mandatory clinical research training for all residents the best means to achieve these desired ends? We stop to question this assumption, reconsider what the main goal of research training in residency ought to be, and offer preliminary suggestions for how to move forward.

## Discussion

This paper is structured around three basic questions: 1) Is mandating original research the answer? 2) What ought to be the central purpose of research training? And 3) What are the alternatives to original clinical research?

### Is mandating original research the answer?

The benefits of resident research curricula have been widely reported.^5^–^8^ Many believe that residents who are successful as researchers in training will be engaged in research as physicians. However, the evidence suggests that producing successful physician scientists involves many more factors.

The successful development of clinician-scientists is influenced by the presence of structured mentoring, a record of past individual achievements such as publications, grant writing and management workshops, manuscript writing workshops, transition programs, career negotiation workshops, and formal programs for career development and career advice.^2^ A systematic review of career choice in academic medicine demonstrated that completion of a graduate degree or research fellowship, the desire to conduct research and participate in an intellectually stimulating environment, stage of training, and gender all influence a clinician’s choice to enter into an academic career.^9^ These studies suggest that the factors that influence a resident’s entry into a research career include – but go well beyond – mere resident research training. Therefore, we need to address multiple factors. We question whether the additional resources needed to comprehensively address the modifiable contributing factors will yield an appropriate return on investment, and from whose perspectives?

### What ought to be the central purpose of research training?

Accepting the centrality of the public interest in medicine and medical education is crucial to our discussion. In Canada, public funding of healthcare, postgraduate medical education and health research carries an implicit social contract to contribute to public welfare. Attempts to ensure equal distribution of benefits arising from health research is politically essential, and a focus on local priorities makes good sense. The Future of Medical Education in Canada Postgraduate project (FMEC PG) clearly invokes the public interest in its first guiding principle: to align the training of physicians with the health and well-being of Canadian patients and communities.^10^ Through consultation with hundreds of stakeholders, “the primacy of medical education’s social accountability role in health care emerged as its core raison-d’être.”^10^ The goal of public funding of healthcare, medical education and research is improved health outcomes for individual patients and communities, accessible health care, and quality service. Since the notion of the public interest must be paramount, we need to ask some hard questions about whether the use of the time, energy, and money spent on resident research training in its most common form, an original clinical research project, yields adequate benefits to patients.

### What are the alternatives to original clinical research?

We know there are efficiencies gained when practicing physicians undertake clinical research, rather than relying only on PhD-trained researchers, because the clinicians are best positioned to ask key clinical questions, to subsequently frame results in a clinically relevant way, and to ensure new knowledge is translated into clinical practice. Should not those individuals who are best positioned to solve society’s health problems, those guardians of public health who have received over a decade of heavily subsidized education and training in medicine, be trained and encouraged to participate in clinical research? And would this not be in the best interest of the public?

The threshold concept from economics – opportunity cost – applies here. Opportunity cost is the lost benefit of not choosing to do some other action with the resources at our disposal. When we deliberate on a particular course of action, we usually explore the advantages and disadvantages of that course of action. Part of our deliberation ought to include a calculation of opportunity cost: what will we fail to gain if we do not spend or invest in *other* opportunities available to us. Applied to the way in which we prepare physicians for research, we need then to consider what might be lost if we engage in the most widely accepted approach during residency – the engagement in original research – or alternatively, we should consider what we could gain if we did something differently.

We believe it would be wise not only to weigh the public benefit and local suitability of this approach compared to others, but to include the opportunity costs, what might be gained by engaging in some other type of training. Our goals might best be met not by having all residents lead original research projects, but by one or more of the alternatives, such as getting them to ask clinical questions that might then be investigated by PhD-trained researchers with resident participation on the research teams. Perhaps undergraduate training should be strengthened, or special residency fellowships instituted, or even the Clinician Investigator Program better utilized. Perhaps we could focus more on all residents learning the quality improvement process that incorporates research into practical and patient-centered system-focused decision-making. Journal clubs and critical appraisal could be expanded. New, so far unimagined, programs might be developed. Residency programs must recognize that spending time and money on original research projects results in losing the benefits of what other approaches to research training might bring. There are hard choices ahead.

Finally we wish to draw attention to the human cost of mandating original clinical research during residency. The literature clearly indicates that a major barrier to conducting research during residency is lack of time.^11^–^13^ This means that there are too many priorities and demands being placed on the residents within the available work week. Burnout and emotional exhaustion have been reported in approximately half of internal medicine residents.^14^ Depression and suicide ideation,^15^ and substance abuse 16,17 have been identified as serious problems with potential for adverse effects on patient care. The FMEC PG report is clear that the health of patients and communities must be prioritized, and that resident fatigue is detrimental to patients.^10^ We must decide what priorities are most important when adding to an already full training load.

On the other hand, small impositions on residents have the potential to dramatically increase the well-being of patients and efficiency of health systems. Stone described the liberty-equality trade-off where small restrictions on some people have the potential to enable major increases of liberty in others.^18^ She writes, “[h]uman freedom can be expanded by society’s willingness to bring problems…under control, sometimes by compelling cooperation in collective endeavors.”^18^ Clinical research study teams have been proposed to get the work done while lightening the load.^19^ The requirement for residents to engage in original clinical research has the potential to make life so much better for so many patients if the resident research makes a breakthrough or the resident goes on to generate a breakthrough during a long and fruitful career in clinical research. Alternately, scholarly work to address local needs may have greater potential to keep patient interests and social welfare at the forefront of our training programs.

## Conclusions

We as a community of medical educators should critically re-think our programs to develop resident researchers. Are they undertaken with the public interest at the center; are they relevant to local needs and supported by the local context? We also need to consider the means by which we achieve those goals. If it is worthwhile to require original research projects during residency, then what are the priorities of local settings that ought to be investigated so as to best serve the public interest? Resident participation in research teams might lead to findings that better serve the public interest and exact less of a toll on residents and on the public purse with the added benefit of providing a better learning environment. We believe that research is important but we question the focus on original resident research, and challenge medical educators to encourage research training aimed steadfastly at the public good.
